# Develop an Adaptive Real-Time Indoor Intrusion Detection System Based on Empirical Analysis of OFDM Subcarriers

**DOI:** 10.3390/s21072287

**Published:** 2021-03-25

**Authors:** Wei Zhuang, Yixian Shen, Lu Li, Chunming Gao, Dong Dai

**Affiliations:** 1 School of Computer and Software, Nanjing University of Information Science & Technology, Nanjing 210044, China; syx@nuist.edu.cn (Y.S.); lilu7qi@163.com (L.L.); 2 Engineering Research Center of Digital Forensics, Ministry of Education, Nanjing 210044, China; 3 School of Engineering & Technology, University of Washington, Tacoma, WA 98402, USA; chunming@uw.edu; 4 School of Cyber Science and Engineering, Southeast University, Nanjing 210096, China; daidong@seu.edu.cn

**Keywords:** device-free detection, indoor intrusion detection, OFDM subcarriers, Wi-Fi sensing

## Abstract

Device-free passive intrusion detection is a promising technology to determine whether moving subjects are present without deploying any specific sensors or devices in the area of interest. With the rapid development of wireless technology, multi-input multi-output (MIMO) and orthogonal frequency-division multiplexing (OFDM) which were originally exploited to improve the stability and bandwidth of Wi-Fi communication, can now support extensive applications such as indoor intrusion detection, patient monitoring, and healthcare monitoring for the elderly. At present, most research works use channel state information (CSI) in the IEEE 802.11n standard to analyze signals and select features. However, there are very limited studies on intrusion detection in real home environments that consider scenarios that include different motion speeds, different numbers of intruders, varying locations of devices, and whether people are present sleeping at home. In this paper, we propose an adaptive real-time indoor intrusion detection system using subcarrier correlation-based features based on the characteristics of narrow frequency spacing of adjacent subcarriers. We propose a link-pair selection algorithm for choosing an optimal link pair as a baseline for subsequent CSI processing. We prototype our system on commercial Wi-Fi devices and compare the overall performance with those of state-of-the-art approaches. The experimental results demonstrate that our system achieves impressive performance regardless of intruder’s motion speeds, number of intruders, non-line-of-sight conditions, and sleeping occupant conditions.

## 1. Introduction

An indoor intrusion detection system (IDS) detects the presence and activity of a human being and serves as an essential component in a diverse range of human–computer interaction applications in the fields of patient monitoring, smart care in homes, and the detection of living people in hazardous environments [1].

Current IDSs have used a variety of specific sensors such as inertial sensors, pressure sensors, ultrasonic sensors, and infrared sensors [2,3,4]. These sensors are deployed in the designated area of the buildings in advance. They collect motion signals in real-time and send out warning messages through predefined threshold mechanisms. Although sensor-based systems obtain motion information directly, these systems’ performances are affected by various factors such as building structure, deployment location, and sensor baseline drift [5,6,7,8,9,10,11].

Another promising technology for intrusion detection is computer vision [12,13,14,15]. A vision-based IDS uses computer vision technology to enable real-time detection of intruders from streaming media information, thus greatly improving its usefulness in surveillance applications. However, vision-based IDSs require image acquisition devices deployed in the environment, and they are prone to be affected by variations of light intensity. Although omni-directional cameras can capture information from all viewing angles in line-of-sight (LOS) environments, they will not work as effectively in non-line-of-sight (NLOS) environments. Furthermore, vision-based IDSs may cause concerns over invasion of user privacy.

Wi-Fi technology, with its wide use and new development, has become a promising technology for IDS. Multiple-input, multiple-output (MIMO) technology is an important and representative Wi-Fi technology that provides high throughput to meet the growing demands for wireless data traffic [16,17,18]. Through orthogonal frequency-division multiplexing (OFDM), MIMO provides channel state information (CSI) at each subcarrier for each transmitter–receiver antenna link pair. The intention of OFDM technology is to reduce the attenuation of wireless signals, which is mainly caused by multipath effects. In recent years, many studies showed that the CSI measurements of subcarriers can be adopted for various sensing applications such as human-presence detection [19,20,21], fall detection [22,23,24], elderly healthcare monitoring [25,26,27], activity recognition [28,29,30], gesture recognition [31,32,33], and human identification and authentication [34]. A Wi-Fi sensing system can be deployed using off-the-shelf commodity Wi-Fi routers and personal computers with the advantage of not requiring additional hardware devices such as sensors or cameras.

**Figure 1:**
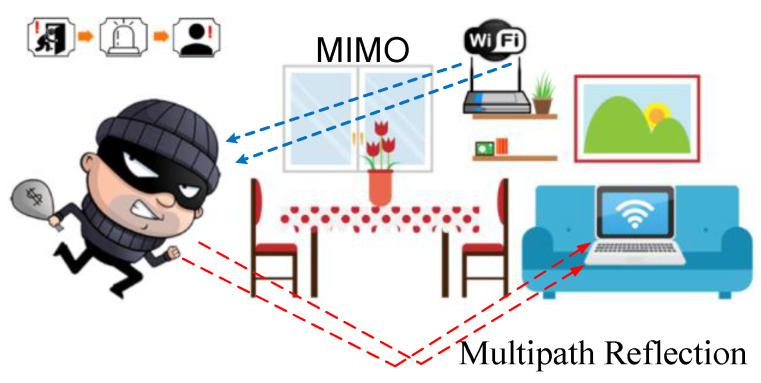
Illustration of detecting indoor intrusion using a commodity Wi-Fi device without additional sensors or cameras.

The key to designing Wi-Fi-based IDSs is to examine and identify characteristic patterns of CSI measurements to extract features and differentiate static (no presence of intruder) and dynamic (with the presence of an intruder) statuses of areas of interest. The illustration image is shown in Figure 1. If the feature patterns selected in the same state are not consistent due to different motion speeds of intruders or different numbers of intruders, the IDS may face severe performance problems [7]. As a matter of fact, a slight motion of a human body will cause a drastic fluctuation of CSI data at the receiver, the location of the Wi-Fi router also affects the strength of multipath attenuation, and even the orientation of the human body will have an impact on CSI measurement values. Therefore, the same status may be correspondent to different feature patterns in different scenarios.

Current device-free Wi-Fi-based IDSs mainly focus on recognizing human presence, while ignoring other important factors such as the number of the intruders, intrusion activity (e.g., slow motion or fast movement), intrusion type (e.g., entry from the window or the door), or intrusion while the owner is sleeping at home. In fact, these are real world intrusion scenarios. In designing an effective system, we must consider these factors, such as the layout of rooms, the placement of furniture, LOS or NLOS, the number of intruders, or whether there are people sleeping in the home. These requirements place tremendous challenges on the design and performance of IDSs.

To address the above challenges, we were motivated to develop an environment-adaptive Wi-Fi-based indoor IDS which can accurately detect intrusion in various real-world scenarios. The system design is inspired by the following preliminary study findings. First, instead of randomly choosing a subcarrier or averaging all MIMO link pairs, we analyzed the sequence signals in each subcarrier and each link pair under different scenarios and found that some link pairs are not sensitive to human motion, nor are their corresponding subcarriers. We proposed an algorithm to select an optimal subcarrier using the three-dimensional CSI packets. Second, we empirically tested that the threshold-based detection method using absolute variance value is not suitable for an adaptive system. Meanwhile, we found that the correlation indicated by maximum eigenvalue or eigenvector is not stable for classifying scenarios as static or dynamic. Based on the theory of OFDM and the Doppler effect, we analyzed the correlation coefficient matrix of time-series subcarriers and found that the correlation coefficient of adjacent subcarriers indicates a pattern statistically similar to the Doppler effect due to the extremely close frequency space. This inspired us to explore potential features from these correlation coefficients to distinguish dynamic scenarios from static.

We implemented our system on commercial Wi-Fi devices and evaluated its performance in real-world environments and in different scenarios. The results showed that our system can accurately detect intrusion with a high average precision of 98.96%, along with a low average false negative rate of 0.73% considering various scenarios such as low motion speed or high motion speed, LOS or NLOS, single intruder or multiple intruders, and whether someone is sleeping at home or not. We also compared the performance of our method against state-of-the-art approaches under different scenarios. We concluded that our proposed method outperforms the compared approaches with higher precision and a lower false negative rate.

Our contributions are summarized as follows:

- We investigated the characteristics of MIMO link pairs and OFDM subcarriers and validated that they are impacted by human motion and it is infeasible to randomly choose a link pair or to average CSI amplitudes of all subcarriers for feature extractions. Then, we designed a link pair selection algorithm to select an optimal link pair for feature extractions.
- We integrated the subcarrier dimension-based features into the classifier based on extremely narrow frequency spacing of adjacent subcarriers and the Doppler effect.
- We implemented our system with commodity Wi-Fi devices and evaluated its performance in real-world scenarios. The experimental results demonstrated our method outperforms both eigenvalue-based and threshold-based methods.

The remainder of this paper is structured as follows. Section 2 introduces the related works of other researchers and explains the main differences between our proposed method and other methods. Section 3 presents the preliminary empirical analysis of the intrusion detection method. Section 4 explains the detailed procedures of system design. Section 5 discusses the evaluation results of our proposed method. Section 6 concludes and presents our future work.

## 2. Related Works

Recently, device-free Wi-Fi CSI-based human activity recognition has attracted a great amount of interest, as it promises to provide a ubiquitous sensing solution using the pervasive Wi-Fi infrastructure. An intelligent detection system called Wi-Vi [19] was proposed in 2013 that enabled small, inexpensive see-through-wall devices that operated in the industrial, scientific, and medical (ISM) band. Gong et al. [10] observed that the detection threshold is correspondent to the level of link sensitivity to human motion and showed that the proposed threshold model achieved comparative detection performance. Palipana et al. [35] provided a model to characterize subcarrier amplitude variation of CSI with human presence that explained the nonlinearity that could occur in CSI under the influence of persons. Qian et al. [21] were the first to incorporate meaningful phase information for device-free human detection by successfully removing the randomness involved in the raw phase. They proposed a novel unified feature using the eigenvalue of the correlation matrix of the CSI. Soltanaghaei et al. [36] proposed an approach for detecting the presence of nonmoving people and provided a single solution to infer home occupancy by using the concept of peripheral Wi-Fi vision. They showed that the system could achieve 96.7% accuracy in occupancy detection with different occupancy scenarios including with an empty location and with moving and stationary subjects. Wu et al. [9] designed and implemented a unified detection approach for stationary persons by modeling and exploiting chest motions of human breathing as an intrinsic indicator of human presence. Zhou et al. [20] proposed a device-free presence-detection and localization algorithm by analyzing CSI fingerprint patterns, and they achieved a presence-detection precision of over 97%. Zhu et al. [37] proposed a novel scheme for robust device-free through-the-wall detection of moving humans with commodity Wi-Fi devices. In particular, they investigated the correlated changes among different subcarriers in the presence of human movement and extracted the mean of first-order differences of eigenvectors of CSI across different subcarriers.

Other CSI-based device-free applications include fall detection, posture recognition, sleep monitoring, driving fatigue monitoring, and even smoking detection. Y. Wang et al. [22] were the first to utilize PHY-layer information with CSI for device-free fall detection in WLANs by implementing WiFall on laptops equipped with commercial 802.11n network interface cards (NICs). H. Wang et al. [23] found a sharp power profile decline pattern in the time-frequency domain indicating a fall and further exploited the complementary characteristics of falls in the time and frequency domains for accurate fall segmentation and detection. X. Liu et al. [38] designed a breath-detection system that could continuously collect the CSI of radio signals and extract breathing patterns. It could track irregular breathing (e.g., sleep apnea) and could also provide breathing information when the person was in different sleeping positions. Jia et al. [39] presented a device-free fatigue detection system, WiFind, to identify the state of drivers by the coefficient of variation of Wi-Fi signals. X. Zheng et al. [40] made the first attempt to develop a ubiquitous passive smoking detection system, Smokey, which could identify smoking activities by analyzing Wi-Fi signals. Zhang et al. [41] took the first attempt to build a ubiquitous passive violence detection system, WiVi, based on the commercial WiFi infrastructure. The system leveraged the correlated features extracted from combined subcarriers, to take full advantage of Channel State Information. These pioneer works have proved that Wi-Fi technology can be used for human motion detection and other applications, whereas our focused study is to further explore features and develop an adaptive IDS for real home environments.

The main differences between our proposed method and other methods are in feature extraction and data preprocessing procedures. Our proposed method introduces a link pair selection algorithm instead of simply calculating the median amplitudes of all link pairs. Some related works, such as [5], selected the distribution of the variance of different subcarriers as the main feature, whereas our work exploits features from correlation information of subcarriers to enhance the generalization ability. Compared with works such as in [6], we select the sum of the correlation coefficients of adjacent subcarriers and the sum of the correlation coefficients of all subcarriers that are greater than a given number ρ instead of the eigenvalue of the correlation matrix of subcarriers. Our goal is to develop a reliable and efficient method to achieve intrusion detection adaptively in real-time.

## 3. Preliminary Empirical Analysis

The key to designing pattern-based approaches is to examine and find characteristic patterns to construct features and differentiate human presence in different scenarios and contexts. We explore the patterns of OFDM subcarriers in static and dynamic environments first, so as to deduce the possible characteristics that can be added to classifiers to distinguish whether or not a human being is present in various scenarios.

### 3.1. MIMO-OFDM Overview

For wireless communications, MIMO-OFDM combines MIMO technology, which transmits different signals over multiple antennas, and OFDM, which utilizes a large number of closely spaced subchannels. CSI describes a communication link where a signal propagates from the transmitter to the receiver. Each CSI entry describes the channel frequency response (CFR) and can be expressed in (1).

(1) $$H=\overset{n}{\underset{i}{\sum }}a_{i}\left(t\right)e^{-j2\pi f\tau_{i}\left(t\right)}$$

In (1), $a_{i}\left(t\right)$ means the amplitude attenuation factor, $\tau_{i}\left(t\right)$ denotes the propagation delay, and f indicates the carrier frequency [1]. The CSI amplitude |H| and phase ∠H are impacted by the indoor objects’ layout, displacement of Wi-Fi devices, and humans.

(2) $$H_{k,m,t}=\left(\begin{array}{ccc} H_{k,1,t} & \ldots & H_{k,1,t+n}\\ \vdots & \ddots & \vdots \\ H_{k,m,t} & \cdots & H_{k,m,t+n} \end{array}\right)$$

**Figure 2:**
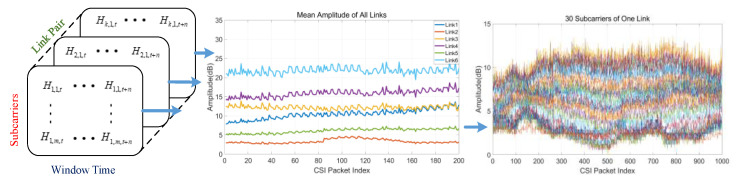
Description of amplitude fluctuation of six multi-input multi-output (MIMO) link pairs and 30 subcarriers based on 3-dimensional mathematical model.

We use sliding time window to monitor the characteristics of a signal sequence. We construct a mathematical model for different subcarriers of multiple link pairs as shown in Equation (2), where $H_{k,m,t}$ means the mth subcarrier’s CFR of the kth link pair during an n time window, as shown in Figure 2. For a typical IEEE 802.11n Wi-Fi device, CSI of each subcarrier of each link pair can be reported using the 802.11n CSI Tool [42], which is the most widely used tool for CSI measurements. Figure 2 shows the mean amplitude of each link pair and instantaneous values of 30 subcarriers of link 5.

### 3.2. Rx-Tx Link Pair Matters

**Figure 3:**
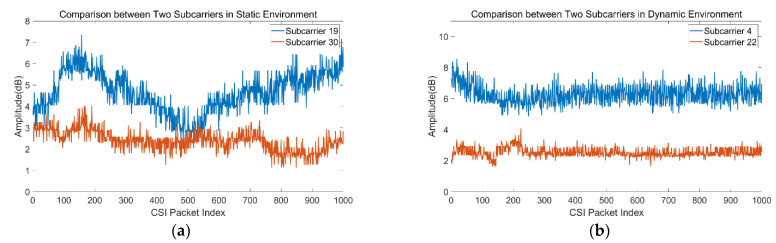
Examples of the fluctuation of subcarriers in static and dynamic environments. (**a**) Link 1 in static environment showing no human presence. (**b**) Link 3 in dynamic environment showing human presence.

Theoretically, the subcarrier information of K Rx-Tx link pairs can be isolated from the data packets of CSI. Existing CSI-based human presence recognition approaches rely either on randomly choosing a link pair or on averaging all subcarriers of one link. Using a typical commercial Wi-Fi device with MIMO from two transmitters and three receivers, we examined the data from various links and subcarriers and found that they perform differently even under the same environment. Figure 3 shows the sequence signals of two subcarriers of link 1 in a static environment and link 3 in a dynamic environment. We can clearly observe the following:

(1) In the static environment, the amplitude variation of some subcarriers is obviously different, with the amplitude variance of some subcarriers being bigger than others.

(2) In the dynamic environment, the amplitude variation of some subcarriers is not obvious and the amplitude is not sensitive to the presence of human beings. Hence it cannot be directly used to extract features.

Therefore, the choice of link pair matters. In our preliminary study, we confirmed that some links are not sensitive to human motion and the corresponding CSI sequence is not informative for identification. In addition, it is impracticable to directly use the threshold method of amplitude or amplitude variance for classification. It can be seen from Figure 3 that even in static environments, some subcarriers showed poor stability.

### 3.3. Adaptive Pattern Matters

As is clear from the above analysis, a simple subcarrier with threshold amplitude or phase mean value, variance, or standard deviation cannot adapt to the environment for static and dynamic differentiation. Consequently, the possible features should be independent of absolute power, since the transmission power parameters will be adjusted according to different situations and thus are scenario dependent, whereas human motion contributes to disturbances of amplitude. Therefore, we choose to analyze the similarity between subcarriers, which could be a good scenario-independent index. The statistical characteristics under this index could be employed as features of the system.

(3) $$COV\left(H_{i},H_{j}\right)=\frac{\sum_{i=1}^{n}\left(H_{i}-{\overset{-}{H}}_{i}\right)\left(H_{j}-{\overset{-}{H}}_{j}\right)}{n}$$

(4) $$COR\left(H_{i},H_{j}\right)=\frac{COV\left(H_{i},H_{j}\right)}{\sigma_{i}\sigma_{j}}$$

Covariance is an index used to express the relationship between variables as shown in (3), where $H_{i},H_{j}$ denote the amplitudes of the i th and j th subcarriers with n packets and ${\bar{H}}_{i},{\bar{H}}_{j}$ denote the expected value of the i th and j th subcarriers. Although covariance can reflect the correlation of two random variables, its value is greatly affected by the dimension, so the similarity of subcarriers cannot be simply represented by the value of covariance. To eliminate the influence of this dimension, we exploit the correlation coefficient to describe it, as shown in (4) where $\sigma_{i},\sigma_{j}$ denote the standard deviation of the i th and j th subcarriers.

**Figure 4:**
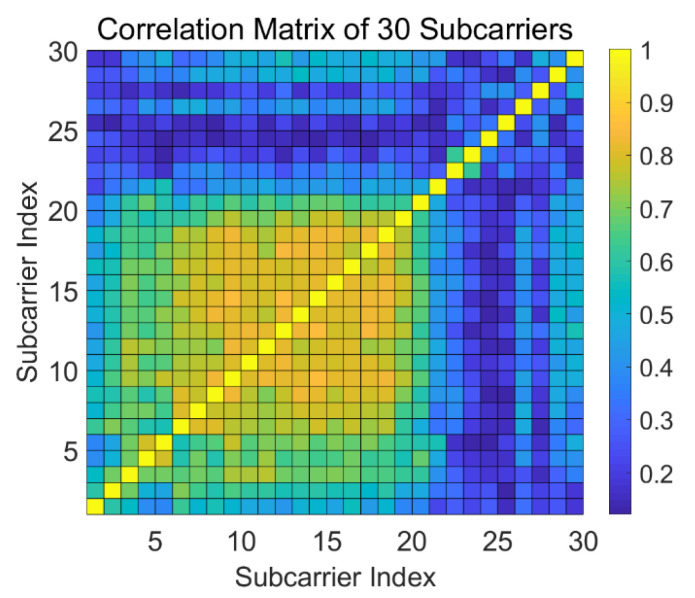
Correlation matrix of 30 subcarriers of a randomly selected link pair during a fixed time sliding window. The color bar represents the entity of each correlation coefficient.

Figure 4 shows the correlation coefficient matrix of two subcarriers from the same link pair at a fixed window, which is a symmetric matrix with order 30 with diagonal entities always being 1. We theoretically assume that with a higher correlation coefficient, the link is more likely to be static and free of intrusion. In contrast, a lower correlation coefficient would probably denote the occurrence of human motion due to the interference of the multipath. To avoid the contamination of correlation features by ambient noise, we reduce the dimension of the matrix by extracting the eigenvalues and eigenvectors under each window to describe the possible features.

(5) Ax=λx

(6) $$\alpha_{i}=\left[\lambda_{i\;max1},\lambda_{i\;max2}\right]$$

(7) $$\nu_{i}=\left[x_{i\;max1},x_{i\;max2}\right]$$

Eigenvalues and eigenvectors naturally reflect the inherent features of matrices. In data mining, eigenvalues are directly used to describe the information contained in the direction of corresponding eigenvectors. To retain as much information as possible in the original signals, we extract the maximum eigenvalue and second largest eigenvalue of the correlation coefficient matrix, as well as the relative eigenvectors. The matrix A in (5) indicates the correlation coefficient matrix of subcarriers, the vector x denotes the eigenvector, and λ represents the eigenvalues. Then, we construct the two-dimension feature candidates as shown in (6) and (7), which represent the eigenvalues and eigenvectors set.

**Figure 5:**
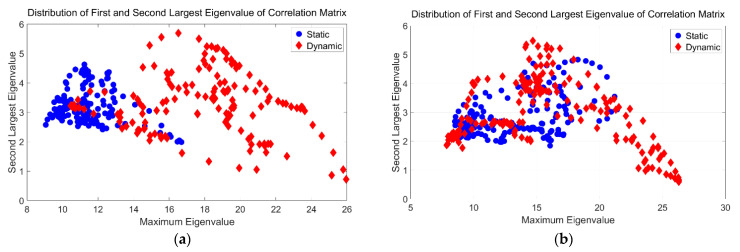
The distribution of first and second-largest eigenvalue of correlation matrix of static and dynamic status under different sliding windows. (**a**) Sample sliding window 1 shows a relatively clear gap between static and dynamic status; (**b**) Sample sliding window 2 shows no clear gap between static and dynamic status.

The preliminary results for classification distinguishing static and dynamic environments are shown in Figure 5. We randomly select two segments of the CSI measurement dataset and then compare the distribution of the two features. From Figure 5a, we can see that there is a relatively obvious gap between the two states. However, the two features in Figure 5b are clearly mixed together, so it is impossible to distinguish the two states. In fact, based on our experimental comparison, the time window of features mixing accounts for more than 56% of the whole dataset, and the preliminary classification results show that the accuracy of classification using the maximum eigenvalue and the secondary eigenvalue is only 48%. Even if the eigenvalue numbers are increased to 10, the highest detection rate can reach only about 88%; at the same time, this approach results in a high false negative rate, which is unacceptable for a real-time detection system.

**Figure 6:**
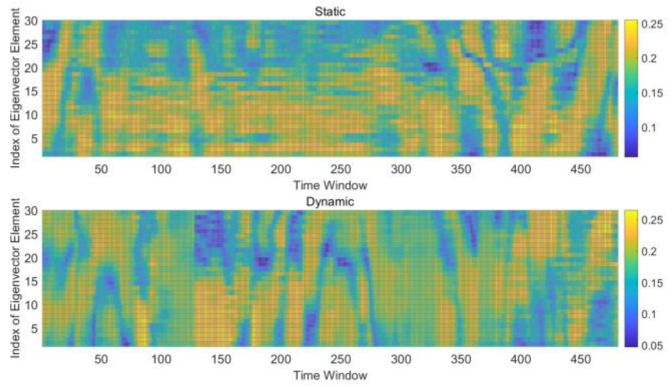
Comparison of distribution of eigenvectors.

Extracting eigenvectors for analysis is essentially the core of principal component analysis, because eigenvectors represent the main components of information. However, Figure 6 shows that the eigenvectors still cannot be used to distinguish the two states. Based on the fact that the correlation does not depend on the environment or transmitting power, we still expect to identify characteristics that can distinguish between the static and dynamic states. Therefore, we studied the time sequence of the correlation coefficient matrix, especially the change process in various scenarios. After random sampling and overall tests, we found a simple and effective feature combination. Details are shown in the next section.

### 3.4. Spatial Patterns Between Subcarriers

Considering the environment and power adaptive capability, the similarity of subcarrier time-series signal variation is still an important index to be further analyzed. Inspired by pioneer researches on Wi-Fi radar and Doppler effect [41,43,44,45], we examined the time-series signals in both the time and frequency domains. A Wi-Fi signal is a type of electromagnetic wave signal, and its wireless link is affected by the speed of the mobile target, so the Doppler frequency shift also applies at the receiver.

**Figure 7:**
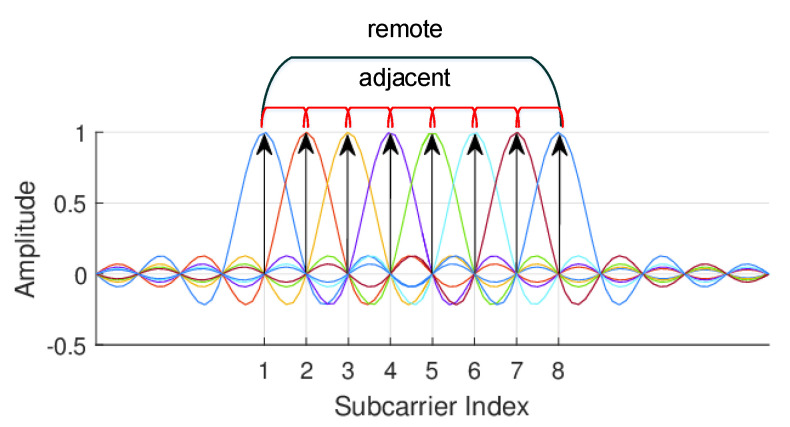
Spectra of orthogonal frequency-division multiplexing (OFDM) subcarriers in frequency domain. For simplifying the description, only 8 consecutive subcarriers are selected.

**Figure 8:**
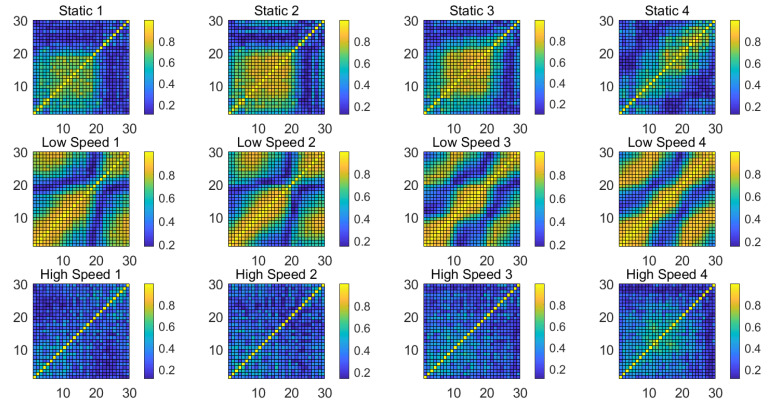
Dynamic distribution of subcarrier correlation matrix of three scenarios including static, dynamic with slow speed and dynamic with high speed using a random sliding window.

Figure 7 shows the spectrum distribution of the subcarriers of OFDM. The carrier frequency spacing is the Nyquist bandwidth, which ensures the maximum frequency utilization. Thanks to the OFDM technology, the frequency difference of adjacent subcarriers is very small, which shows that the Doppler frequency shift has a high similarity concurrently mapping to the sequence packets after the recovery of the analytical signal; in contrast, the frequency difference of subcarriers far away from each other is large, and the impact of moving targets on that difference is great. Based on this theoretical deduction, we randomly select the subcarrier correlation coefficients with equal length windows under different scenarios shown in Figure 8, which demonstrate the following patterns:

In a static state, the correlation coefficients of some adjacent subcarriers are high, and the region with high correlation coefficients is always moving.

In a dynamic state with low motion speed, the correlation coefficients of adjacent subcarriers are very high on the overall view, which are widely distributed, more stable, and larger than those in the static state.

In a dynamic state with high motion speed, the correlation coefficients of adjacent subcarriers are smaller and more stable, which are smaller than those of a dynamic state with low motion speed and static state.

In general, the number of high correlation coefficients in a dynamic state with slow-motion speed is greater and is stable, the number of high correlation coefficients in a dynamic state with high motion speed is the least and is stable, and the number of high correlation coefficients in a static state is medium but not stable.

Based on the above observation, we proposed two possible feature variables in a mathematical model: (1) the sum of the correlation coefficients of adjacent subcarriers and (2) the sum of the correlation coefficients of all subcarriers that are greater than ρ. The first feature variable mainly describes the details, whereas the second mainly describes the general view.

**Figure 9:**
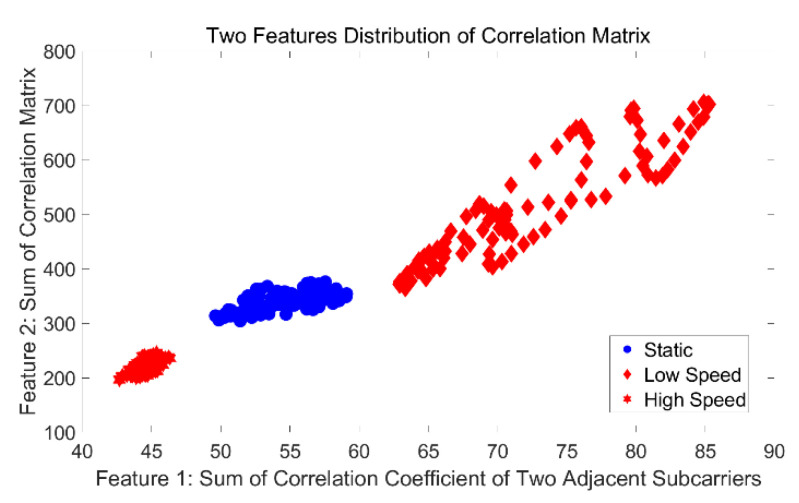
The distribution of our proposed features for classifying static, low speed, and high speed.

To preliminarily test whether the selected feature variables distinguish well between static and dynamic states, we preprocess the signals using our method in the next section and generate the distributions of features under multiple windows. Figure 9 shows the distribution of proposed features under random windows with ρ  equal to 0.7. We can observe that there is a clear gap between static and dynamic states.

## 4. System Design

**Figure 10:**
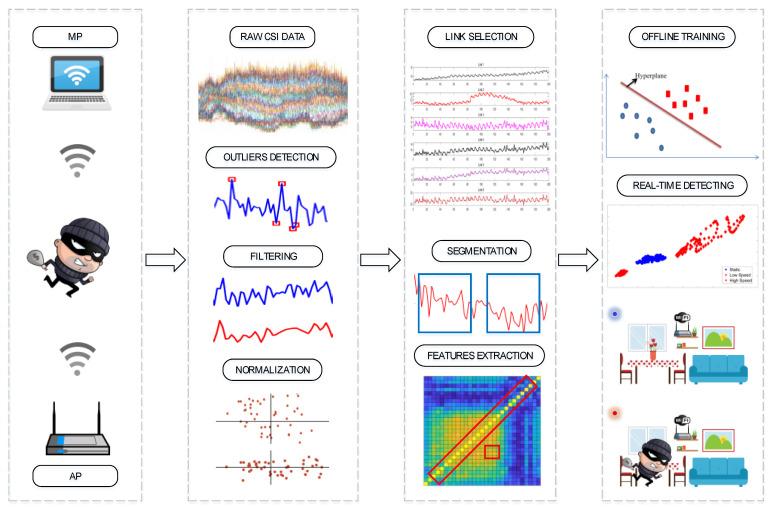
Main diagram of our proposed scheme.

The intention of the proposed system is to adaptively detect indoor intrusion in various intrusion scenarios. Based on the empirical analysis, we designed the architecture of our system. As shown in Figure 10, we first built a simple CSI acquisition system based on 2 × 3 MIMO, in which the network card is compatible with the 802.11n protocol. Due to the influence of environmental noise and wireless interference, the collected CSI packets often contain outliers that can readily have a negative impact on system performance, so these outliers must be eliminated. After wavelet de-noising and normalization, the subcarrier amplitudes are adjusted to a fixed upper and lower range. Then, we designed an algorithm to select the appropriate link from which to extract the two correlation coefficient features. By setting the short step sliding time window mechanism, we divided the data packet into several data segments to support vector machine (SVM) model off-line training and performance testing [46,47,48,49,50]. Finally, the system was set for real-time detection with the appropriate model parameters.

### 4.1. CSI Raw Data Gathering

Daniel Halperin et al. [42] constructed the CSI tool, elaborating the measurement and processing methods of CSI in a Wi-Fi signal. Our system uses a commercial router as the Access Point (AP) and a laptop equipped with an Intel 802.11n compatible NIC as the Monitor Point (MP) to build the hardware platform. It collects the indoor CSI signal with the help of CSI Tool. In the Ubuntu 12.04 operating system, the MP can continuously receive CSI packets from the wireless router using ping operations.

Each CSI packet contains seven data fields. The specific meanings of these seven fields are shown in Table 1. Each data element in the CSI structure is described in the complex signal model, which reflects the amplitude and phase information of its channel state. The amplitude, phase, and other features represented in the CSI packet are adopted as the raw data in the subsequent data processing.

### 4.2. Preprocessing

Three data preprocessing modules are described in this section, which are used respectively for outlier detection, wavelet-based filtering, and normalization, with the goal of having the results minimally affected by varying scenarios.

#### 4.2.1. Outlier Detection

The purpose of outlier detection is to detect abnormal changes in the received CSI data. To ensure the stability of quality of service (QoS) in different environments, the transmitter automatically adjusts the radio transmission power, which is reflected in the fact that the CSI values in the time domain include some outliers.

In this module, the abnormal data can be identified by the local outlier factor (LOF), which is used to identify abnormal conditions received by the MP. The LOF algorithm determines the anomaly degree of a sample by calculating its local reachable density. Generally, the LOF values of the data in the cluster are close to 1, whereas the LOF values of the data at the edge of the cluster are slightly larger than 1. Accordingly, those data with LOF values far greater than 1 may be the outliers that need to be eliminated in the system. The algorithm is as follows:

(1) Input the preliminary dataset  D and the threshold value of outlier factor ξ. The preliminary dataset  D can be obtained from the raw-data gathering module, and the outlier factor ξ can be set according to the experimental comparisons.

(2) Calculate the local reachable density of each object using (8). The local reachable density of object *p* can be represented as one divided by the average accessible distance between object *p* and $N_{k}\left(p\right)$.

(8) $$lrd_{k}\left(p\right)=1/\left[\frac{\sum_{o\in N_{k}\left(p\right)}reach-dist_{k}\left(p,o\right)}{\left|N_{k}\left(p\right)\right|}\right]$$

where, $reach-dist_{k}\left(p,o\right)$ denotes the reachability distance between object p and o, $N_{k}\left(p\right)$ denotes the set of k nearest neighbors of object p, and $\left|N_{k}\left(p\right)\right|$ denotes the number of elements in $N_{k}\left(p\right)$. The reachability distance between object p and o is defined as $reach-dist_{k}\left(p,o\right)=max\left\{k-dist\left(o\right),d\left(p,o\right)\right\}$, where, k−dist(o) denotes the Euclidean distance of the object o to the k-th nearest neighbor, and d(p,o) denotes the Euclidean distance between p and o.

(3) Calculate local outlier factor $LOF_{k}\left(p\right)$ of each object using (9). The LOF of object p represents the degree of abnormality of  p. The value of LOF is proportional to the probability that the object is abnormal.

(9) $$LOF_{k}\left(p\right)=\frac{\sum_{o\in N_{k}\left(p\right)}\frac{lrd\left(o\right)}{lrd\left(p\right)}}{k}$$

**Figure 11:**
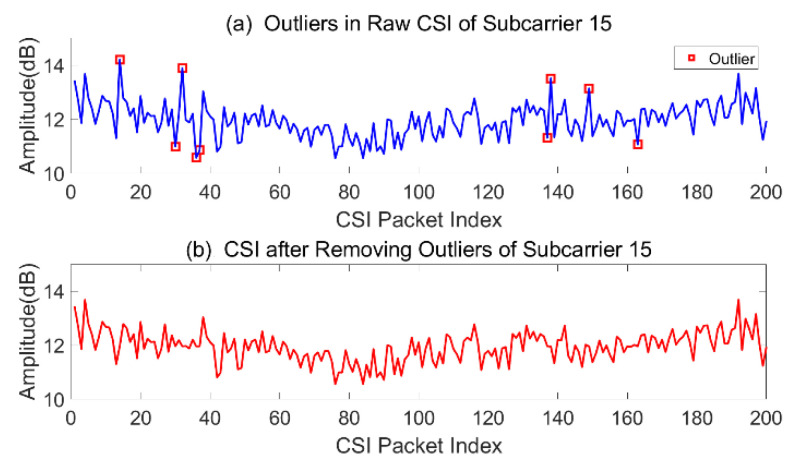
Detection and elimination of outliers.

(4) Filter outliers and output the final sanitized dataset. If an object’s LOF value is far beyond the threshold factor ξ, these data will be eliminated, and a new dataset will be established. Figure 11 shows the raw sequence signals with outliers and processed signals without outliers.

#### 4.2.2. Wavelet-Based Filtering

The CSI time-series signal collected in our system is a typical time-varying nonstationary signal, which contains Gaussian white noise or power-line interference. Therefore, it is necessary to exploit a signal de-noising tool that not only eliminates the high-frequency noise but also retains the human-motion components hidden in the high-frequency part. The discrete wavelet transformation (DWT) de-noising method gives both fine frequency resolution for low-frequency signals and time resolution for high-frequency signals. The output of DWT can be fed to a wavelet filter to remove noise while preserving human motion information in different scenarios [1].

The wavelet basis and decomposition level, the threshold rule, and the threshold function are the key factors that affect the final de-noising effect. In our study, human-motion-related signal frequencies range from 0 to 50 Hz for all scenarios. Based on experimental comparisons and pioneer studies on nonstationary signals, we finally chose the db-8 wavelet (the Daubechies) with two decomposition levels, which has been shown to give better performance in electrocardiogram signal analysis [2].

**Figure 12:**
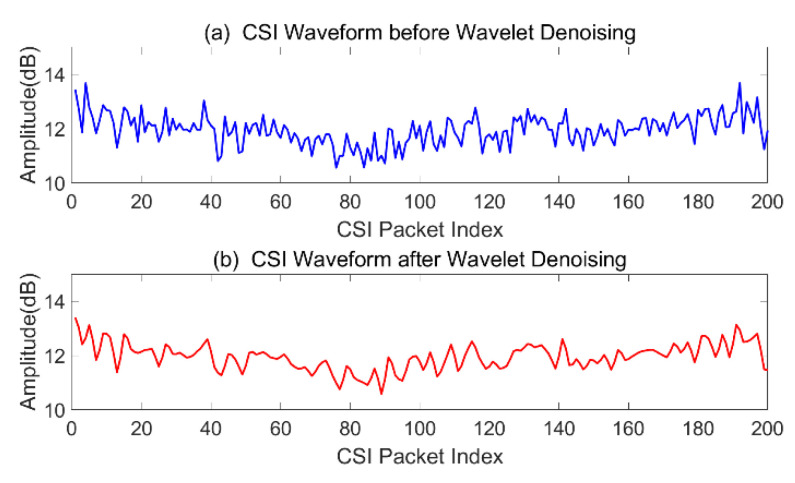
Discrete wavelet transformation (DWT)-based CSI signals de-noising.

To preserve the signal details as much as possible, we select *rigorous* and *soft thresholding* so as to retain more features within the signals corresponding to the detail coefficients. After thresholding the detail coefficients, the de-noised CSI signals are reconstructed using new detail coefficients and all level approximations. A comparison of original and de-noised CSI signals is illustrated in Figure 12.

#### 4.2.3. Normalization

Due to adjustments in transmitting power and the QoS-center mechanism, the raw CSI amplitudes vary in different environments and scenarios. CSI data must be standardized to the same range for later correlation analysis and feature extraction, so we exploit the min–max normalization method to map the data into [−1, 1].

(10) $$x^{\prime }=\left(x-min\right)/\left(max-min\right)$$

**Figure 13:**
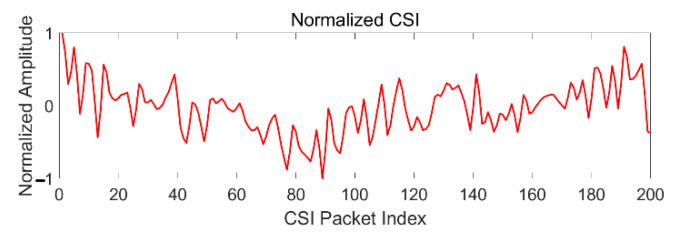
Normalization of CSI after de-noising.

After wavelet de-noising, the maximum and minimum amplitudes in a fixed window are calculated, then the discrete *x* is transformed into a new value $x^{\prime }$ according to (10). The unit limitation on data is removed, and they are transformed into a dimensionless pure value, while the correlation between subcarriers is preserved. The normalized CSI signal is shown in Figure 13.

### 4.3. Link Pair Selection

Based on empirical analysis of OFDM subcarriers, we conclude that random selection of a link pair is unreliable for feature extraction, which will tremendously affect the final classification performance. According to experimental results, the sensitivity of the variance of subcarrier correlation coefficients to human motion varies, thus in either a static or dynamic environment, the stability of the change in the subcarrier correlation coefficients is critical and directly affects the precision and the false negative rate. We analyze the stability of *K* links (*k* = 6 in this system) and output the optimal estimation using the following mathematical model:

(11) $${\hat{X}}^{k}{}_{ij}=var\left(A_{i,j}^{k}\right),\left(\forall i,j\in \left[1,m\right],i<j\right)$$

(12) $$K_{opt}=argminE\left({\hat{X}}^{k}{}_{ij}\right),\left(k\in \left[1,K\right]\right)$$

where $A_{i,j}$ indicates the ij th entry of correlation coefficient matrix A of the k-th link pair, m is the number of subcarriers which equals to 30 in our system, and K  denotes the total number of link pairs which equals to 6. Since the correlation matrix A is a symmetric matrix and the entries on the diagonal are all 1, we count only one-half of the matrix entries. Equation (11) outputs the variance vector ${\hat{X}}^{k}{}_{ij}\;$ that will be considered a set of random variables as an input into (12). Then, we calculate the expectation of the ${\hat{X}}^{k}{}_{ij}$ and output the optimal index of link pair $K_{opt}\;$ related to the minimum expectation. The subcarriers of the selected link pair will then be adopted for feature extraction.

### 4.4. Features Extraction

**Figure 14:**
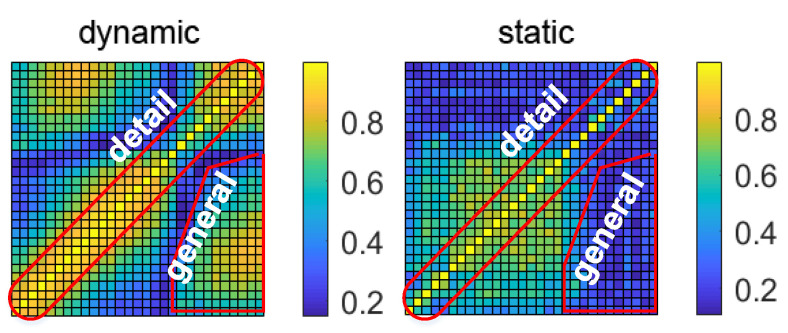
The interest area for extracting detail and general features.

Based on the subcarrier analysis above, we conclude that because of the similarity of Doppler frequency shift between adjacent subcarriers, we can deduce the characteristics from the distribution of correlation coefficients of subcarriers. The red area in Figure 14 shows where our features are located: the detail part denotes the correlation coefficient of adjacent subcarriers, and the general part describes the correlation coefficients that are greater than a fixed threshold.

We describe two previously proposed features in mathematical models in Equations (13) and (14): (1) the sum of the correlation coefficients of adjacent subcarriers and (2) the sum of the correlation coefficients of all subcarriers that are greater than ρ.

(13) $$D=\overset{m}{\underset{i,j=1}{\sum }}A_{i,j},(\forall i,j\in \left[1,m\right],j-i=1)$$

(14) $$G=\overset{m}{\underset{i,j=1}{\sum }}A_{i,j}>\rho ,\left(\forall i,j\in \left[1,m\right],i<j\right)$$

(15) $$D_{w}=\left[D_{1},\cdots ,D_{i},\cdots ,D_{n}\right]$$

(16) $$G_{w}=\left[G_{1},\cdots ,G_{i},\cdots ,G_{n}\right]$$

where m means the total number of subcarriers, which equals 30 in our system, $A_{i,j}$ with constrained i,j denotes the correlation coefficient of each adjacent subcarrier pair, and ρ  defines a threshold for detecting the high coefficient of all subcarriers which has an impact on classification performance. Two features are calculated and then stored in D  and  G. For each fixed window  w, we continuously sample the two features and generate two datasets, $D_{w}\;$ and $G_{w}$, as expressed in Equations (15) and (16), for training and testing in the future. We did not add the CSI phase features into our current system, mainly because the phase must be calibrated and sanitized which will increase calculation complexity and affect the valuable bandwidth. Therefore, we will exploit the phase information for other applications in our future works, as the extracted correlation coefficient features have shown satisfactory performance.

### 4.5. Real-Time Segmentation

**Figure 15:**
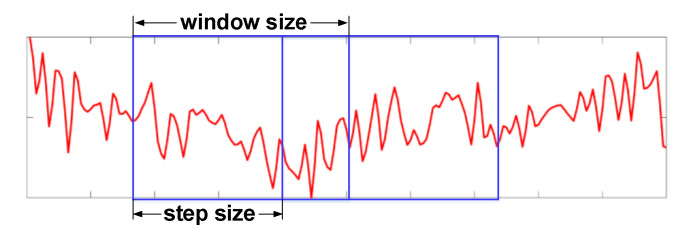
Segmentation for subcarriers using a fixed window and step size.

For the real-time monitoring system, data windowing is critical for building training and testing sets. When the window is smaller, there will be more feature sets in the samples, and the detection sensitivity is higher; but at the same time, the generalization ability of the machine-learning model gets worse, thus making overfitting more likely. By contrast, when the window is larger, the adaptability of the model is stronger and less likely to overfit, but it is easier to lose patterns and thus becoming underfitted. Therefore, it is necessary to leverage the window and step size based on recognition performance. In our experiments, we found that when the window size is 2 s and the step length is 0.1 s, i.e., when the data overlap rate is 95%, we can achieve a high recognition rate as well as quickly detect an intruder within only 2 s. The illustration diagram is shown in Figure 15.

### 4.6. Classifier

Because the features were manually extracted from the raw CSI and only binary classification is required, we choose a shallow machine-learning model to identify static and dynamic states. A shallow machine-learning model does not need massive amounts of data and complex computation resources in the way a deep-learning model does. Shallow-learning algorithms, such as *k* nearest neighbors, SVM, and self-organizing map algorithms, are widely used for detection and recognition applications. SVM separates data points using a set of hyperplanes in high-dimensional space to maximize the functional margin: that is, the distance to the nearest training data points of any class [1]. It can also use a kernel function to classify classes that are not linearly separable [2].

(17) $$T=\left(f_{1},l_{1}\right),\left(f_{2},l_{2}\right),\cdots ,\left(f_{n},l_{n}\right)$$

(18) $$\underset{w,b,\varepsilon_{i}}{min}\frac{1}{2}\|w{\|}^{2}+C\overset{n}{\underset{i=1}{\sum }}\psi_{i},\psi_{i}\geq 0,s.t.\;l_{i}\left(w\cdot l_{i}-b\right)\geq 1-\psi_{i},\forall \left(f_{i},l_{i}\right)\in T$$

Given a training dataset T as shown in (17), where $f_{i\;}$ is a two-dimensional feature vector, $l_{i}$ is the label for static and dynamic state, the SVM algorithm will process the optimization problem of (18), where $\psi_{i}$ denotes the misclassification degree, and C is a parameter needing to be tuned that determines the tradeoff between the margin size and the amount of error in training.

We exploit the radial basis function (RBF) kernel in our SVM model, which has been proven to have better performance. Cross-validation was also conducted to search for the optimal parameter combination, c,g, that can reflect the fitting degree of the model for unknown test data more accurately and objectively. Through experimental comparisons, we conclude that the system outputs an overall accuracy of 98.96% with the optimal parameters *c* being 8.0 and *g* being 0.03125. In the next section, we evaluate our proposed system and discuss its classification and detection performance.

## 5. Evaluation

In this section, we first introduce the experimental evaluation environment and the prototype of the experimental platform. We then discuss the impact of environments and scenarios on performance. Finally, we compare the classification performance of our system with those of the state-of-the-art approaches.

### 5.1. Experimental Setup

**Figure 16:**
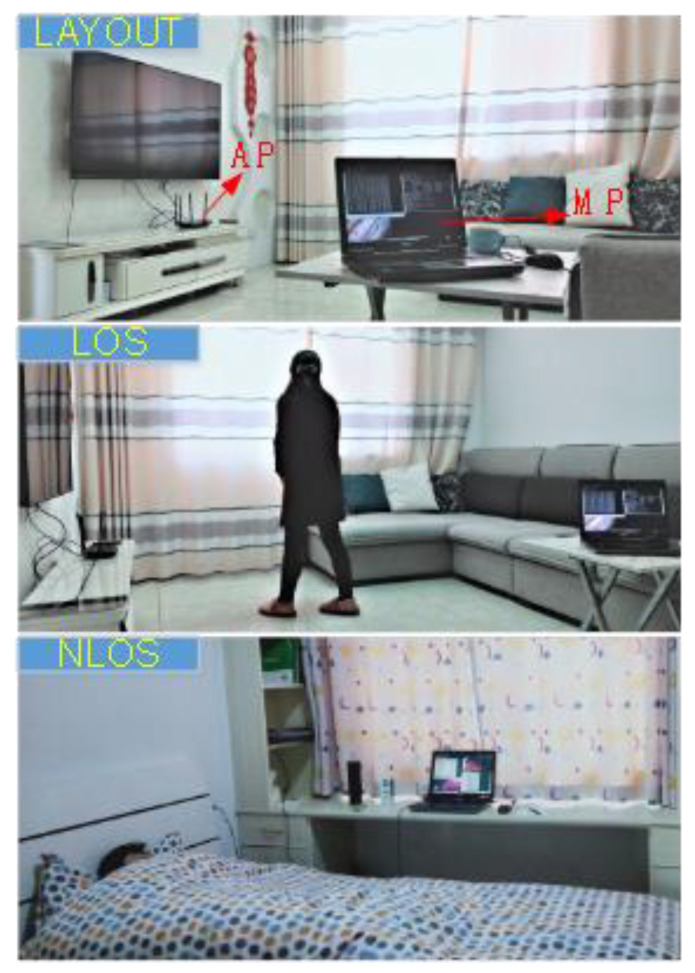
The interest area for extracting features.

**Figure 17:**
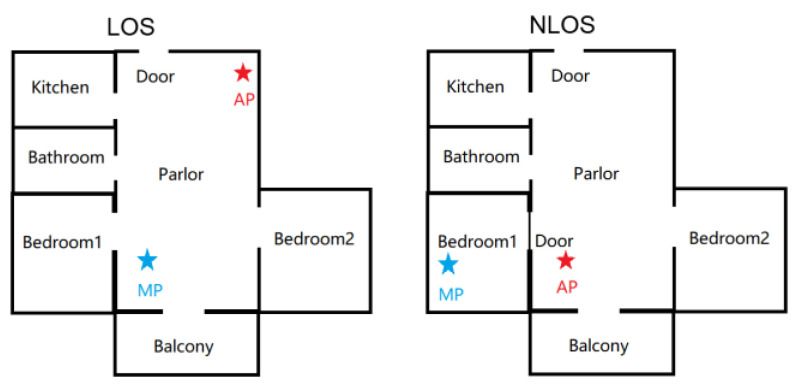
The layouts of selected apartments with different deployment of access point (AP) and monitor point (MP).

To simulate real-world intrusion scenarios as much as possible, we first chose a real apartment as the experimental site, an approach that differs from others’ research works that used a laboratory as the experimental site. Figure 16 shows the selected apartment for data gathering and testing. AP and MP were deployed as shown in Figure 17. The “intruder” walked randomly into the living room with different speeds, including slow (approximately 0.5 m/s) and fast (approximately 2 m/s). We next designed a scenario with multiple intruders and someone sleeping in the bedroom. To the best of our knowledge, this is the first time such a scenario has been considered in CSI-based intrusion detection.

Unlike most studies, which considered only a single intruder, we intended to verify whether our system can provide stable performance with multiple intruders. In addition, we considered whether a person turning over in bed and normal breathing during sleeping would affect the detection performance. Finally, we compared the detection performance of the through-the-wall environment by deploying the AP and MP in different rooms, separated by a 20-cm-thick concrete wall, which is also a typical layout in the real home environment. All experimental scenarios are shown in Table 2.

We implemented our system with commodity Wi-Fi devices and evaluated its performance in the previously described scenarios. We used a TL-WDR5620 wireless router equipped with two antennas as the AP which was deployed on an office desk in the living room. We adopted the Dell Latitude D360 laptop with an Intel Wi-Fi Link 5300 card as the MP, which was placed in the living room for LOS test and in the bedroom for NLOS test. We set the AP to operate in IEEE 802.11n mode at 2.4 GHz with 20-MHz bandwidth and set the MP to run Linux Ubuntu 10.04 with CSI Tool installed. In our experiment, the CSI sampling rate is 50 Hz, i.e., 50 packets per second from the AP. The sampling duration of each group was set at 50 s, so the sample size of each group is 2500 packets. Each group collection run was repeated five times. We measured the indoor temperature of the experiment as 20 °C (68° F) and the outdoor temperature as 8 °C (46.4° F). The environment surrounding the residence was quiet without human activities. After training the SVM model, we imported the test data into the model and obtained four types of outputs: true positive (TP) where there is human presence and the system reports so, false positive (FP) where there is no human presence but the system reports human presence, true negative (TN) where there is no human presence and the system reports correctly, and false negative (FN) where there is human presence but the system does not report it. For a real-time indoor intrusion detection system, we are more concerned about two indicators, TP and FN. The former represents the number of samples detected when an intrusion occurs, and the latter represents the model failing to output a dynamic state when an intrusion happens. To show the system performance more clearly, we define the following indicators:

(19) Precision=TP/(TP+FP),FNR=FN/(TP+FN)

Precision indicates what percent of the reported dynamic cases are indeed dynamic, whereas false negative rate (FNR) means what percent of dynamic cases are failed to be detected. We examined these indicators to evaluate the performance of the system in various scenarios, as described in the following sections.

### 5.2. Overall Performance

**Figure 18:**
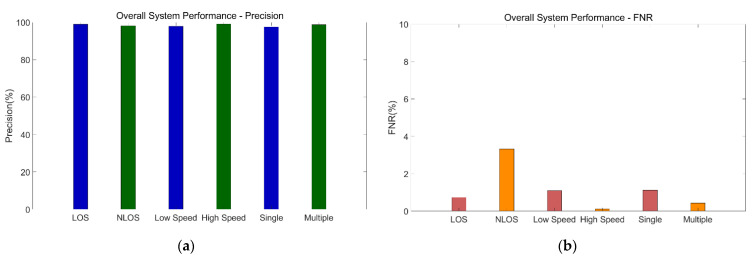
Overall system performance of precision and false negative rate (FNR). (**a**) Precision. (**b**) FNR.

We would like to make direct comparisons with other approaches. However, the experimental sites used in testing the state-of-the-art methods in other studies are almost in laboratories, and many scenarios are not considered. We therefore cannot make direct comparisons for these scenarios. Thus, we first present the overall evaluation of our system’s performance, and we then compare the results with those generated from two other state-of-the-art methods. Figure 18 shows the overall performance of our system.

First, we average the results of five tests for each group and calculate the performance in different scenarios. Under LOS, the system’s average precision can reach 98.96%, and the average false negative rate (FNR) can be as low as 0.73%, which means that the possibility of erroneous classification of an intrusion is very small, and the system is robust. Even in NLOS, the average precision can reach 98.17%, and the average FNR is 3.32%, which is slightly higher than that in the LOS environment, indicating that in the through-the-wall environment, it is relatively easy to misclassify. Under the conditions of high motion speed and low motion speed, the average precision values are 97.94% and 99.09% respectively. The average precision values with one person and multiple persons are 97.53% and 99.02% respectively, all with low FNRs. In general, scenario differences have little impact on the performance of our proposed system, which performs well in the real-world environment. The performance evaluation is detailed in the following sections.

### 5.3. Impact of Sliding Time Window Size

We need to evaluate the impact of the sliding time window size of the samples on the performance of the proposed model, since the window size is critical for feature generalizability, for the system computation needs, and for the response time. We try to find the optimal window size that balances recognition rate and system response time.

**Figure 19:**
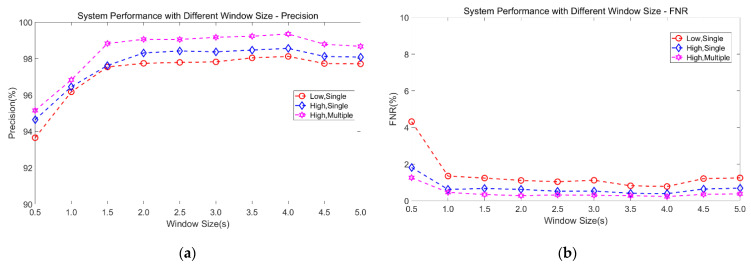
System performance with different window size. (**a**) Precision. (**b**) FNR.

Figure 19 shows the impact of different window sizes on precision and FNR. It can be clearly observed that as the sliding time window becomes larger, the precision increases and the FNR reduces correspondingly until the size is 4 s, which means that when the window is larger than 4 s, system performance begins to decline. This indicates that when the window size is enlarged, with a corresponding decrease in the number of samples, the SVM model underfits, and its sensitivity to features decreases. At the same time, as window size increases from 2 s to 4 s, although precision increases, the average growth rate in precision is not high, at only 0.14%. Therefore, to improve the model’s generalization ability, i.e., the ability to accurately classify features, we set the window size to 2s which brings average precision of 98.38% and an average FNR of 0.67%.

### 5.4. Impact of Speed and Number of Intruders

The motion speed of indoor intruders is not constant. We must evaluate the impact of different speeds on performance, as well as the impact of a single intruder or multiple intruders. According to our empirical analysis, since the features we choose are derived from the correlation of subcarriers, in which the correlation coefficient of adjacent subcarriers is very sensitive to the motion speed, thus we can differentiate the low and high speeds.

**Figure 20:**
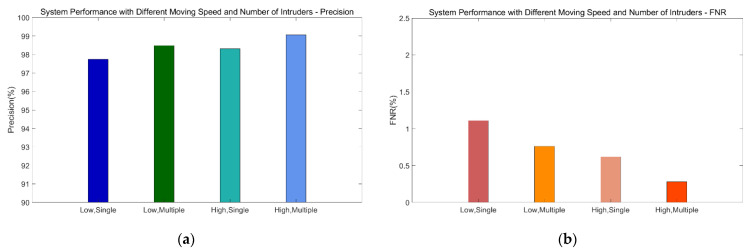
System performance with different speed and intruders. (**a**) Precision. (**b**) FNR.

Figure 20 shows the impact of different motion speeds and numbers of intruders on performance. The precision of the system is lowest (97.74%) and the FNR is highest (1.11%) with a single person moving at low speed; the precision is highest (99.06%) and the FNR is lowest (0.28%) with multiple persons moving at high speed.

Compared with the low-speed detection results from other works, our system has better performance detecting low-speed movement. Generally, the lower the speed, the more difficult to detect; and the more people there are, the higher the detection accuracy is. If we consider our MIMO system as a Doppler radar, the larger the moving target is, the easier it will be to detect it, which is consistent with the theoretical analysis. Furthermore, our experimental results inspire us to apply our system for speed detection in the future work.

### 5.5. Impact of Sleeping and Respiration

**Figure 21:**
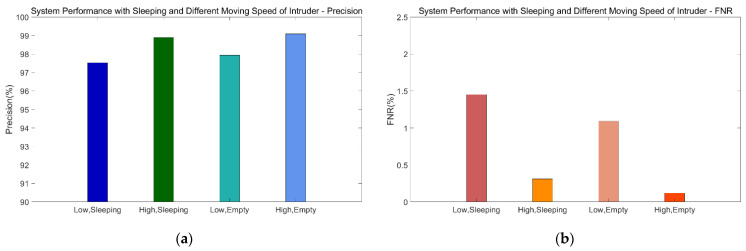
System performance in sleeping scenario. (**a**) Precision. (**b**) FNR.

In addition to the impact of the number of intruders on performance, we also evaluate the system’s ability to recognize intrusion when someone is sleeping at home and the impact on system performance of a body turning over and of breathing. We add the feature set of body rotation and normal breathing into the static data, select CSI data for different speeds from the dynamic dataset, and then rebuild the SVM model. Figure 21 shows the system performance when someone is sleeping at home.

When the intruder’s speed is low, even if someone at home is resting, the precision given by the system can still be as high as 97.52%, which is only 0.42% lower than that in a non-sleeping situation. FNR is as low as 1.45%, which is 0.36% higher than that in a non-sleeping situation.

In the situation of fast movement of intruders, the precision can reach 98.89%, and FNR is only 0.31%. The results show that our approach has great adaptability to sleeping situations; even if there are slight body movements and normal breathing, the impact on recognition rate is relatively low.

### 5.6. Comparison with STATE-OF-THE-ART Methods

We discussed the performance of our proposed method in various scenarios. To verify the advantages of the proposed method, we need to compare it with other intrusion detection systems. Because the experimental sites adopted in most research are in laboratories rather than in real home environments, and because almost no one has considered the scenarios of multiple intruders and with somebody sleeping at home, we compare our method against the most widely used time-sequence human detection methods applied in the concerned scenarios. Specifically, we compare our proposed method with the eigenvalue-based method used in PADS [6] and the variance-based method used in SIED [5], which are two commonly used approaches at present. In addition to the above two studies, there are also other studies such as [35,51,52,53] using similar features. In PADS, the authors exploited the amplitude and calibration phase of CSI to detect human movement. By calculating the correlation matrix of amplitude and phase from continuous CSI measurement, PADS derived the maximum eigenvalues of two matrices for mobile human detection for classification. In SIED, the authors used the patterns of distribution of the variance and a hidden Markov model to identify moving objects. After preprocessing the raw CSI data, we extracted the relevant features used in PADS and SIED, trained each SVM model, and then examined the performance.

**Figure 22:**
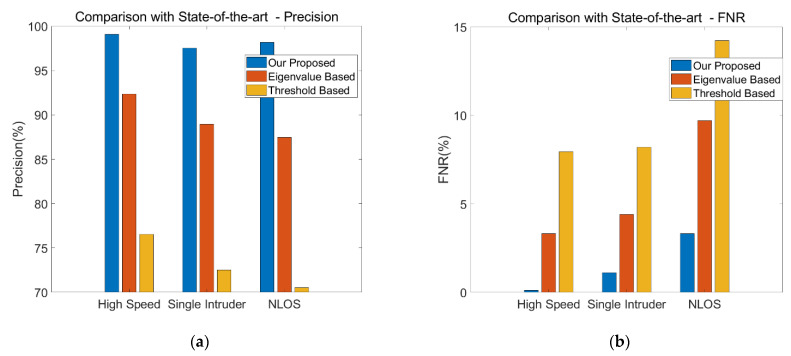
Comparison with other methods. (**a**) Precision. (**b**) FNR.

Figure 22 plots the comparison results for precision and FNR for the three methods using different features. Three scenarios are considered, including single intruder with high speed, single intruder with low speed, and NLOS. The first two are based on the average values in an LOS environment, and the latter is based on the average values of high speed and low speed in an NLOS environment. Note that for the eigenvalue-based method, if only the maximum eigenvalue and the second largest eigenvalue of the correlation coefficient matrix are selected as the features, the precision and FNR are very poor. Therefore, to reflect the maximum potential of this method, we select the top 10 maximum eigenvalues as the features, which is an option that outputs the optimal performance. Meanwhile, the threshold-based method uses the distribution of CSI amplitude variance as the feature for training.

It can be clearly observed that our proposed method outperforms both eigenvalue-based and threshold-based methods for all selected scenarios. For the most common single person intrusion, the precision of our method can achieve 97.53%, whereas the precision of the eigenvalue-based method is 88.95%, and the threshold-based method has the worst performance, at only 72.53%. In addition, in the complicated NLOS environment, our method can achieve the precision of 98.17%, which is 10.72% higher than that of the eigenvalue-based method and 27.66% higher than that of the threshold-based method. Moreover, one of the advantages of our method is that its FNR is far lower than those of the other two methods in the same scenarios, which is critical for a real-time detection system. This result is expected, since our proposed system only relies on the correlation of adjacent subcarriers and the high coefficient region, regardless of differences in received signal strength, transmitting power, and environment variables. This demonstrates that our method is more adaptive to various environments.

## 6. Conclusions and Future Work

In this study, we developed an environment- and scenario-adaptive indoor intrusion detection system with commodity Wi-Fi devices. We first investigated the characteristics of MIMO link pairs and OFDM subcarriers impacted by human motion and validated the infeasibility of randomly choosing a link pair or of averaging CSI amplitudes of all subcarriers. Based on these findings, we designed an optimal link-pair selection algorithm as the first step of developing our system. Since only one link pair is selected, the number of antennas will not affect the performance. Using the theory of narrow frequency spacing of adjacent subcarriers and the Doppler effect, we integrated the subcarrier dimension-based features into our classifier and trained an SVM model to detect the static and dynamic states. We implemented our system with commodity Wi-Fi devices and evaluated its performance for various scenarios, including low speed and high speed for intruder motion, LOS and NLOS, single intruder and multiple intruders, and scenarios in which someone is sleeping at home. A sliding window size of 2 s is selected in our system. With the running time of the detection algorithm on an ordinary computer being negligible, it will take 2 s to detect the intrusion in both LOS and NLOS environments, which means our method can be applied in real-time. The experimental results demonstrated that our system performs well in terms of precision and FNR, and outperforms both eigenvalue-based and threshold-based methods. Furthermore, we verified that the correlation coefficients of adjacent subcarriers are highly sensitive to human movement, which inspires us to consider applying our method to Wi-Fi-based speed detection and crowd counting in the future. With the development of Wi-Fi 6 standard, we also consider extending our system to support the next generation Wi-Fi 6 IEEE 802.11ax specification.

## Figures and Tables

**Table 1:** Channel state information (CSI) data field table.

Parameter	Indication
Timestamp_low	Low 32 bits of network card used with 1 MHz clock
Nrx	Number of antennas of the receiver
Ntx	Number of antennas of the transmitter
RSSI	RSSI value measured by the receiver
Noise	Channel noise
rate	Sampling frequency
csi	CSI value

**Table 2:** Experimental Scenarios and Grouping.

No	Placement	Speed	Intruders	Sleeping
1	LOS	Null	0	No
2	LOS	Slow	1	No
3	LOS	Fast	1	No
4	LOS	Slow	2	No
5	LOS	Fast	2	No
6	LOS	Null	0	Yes, with turning over
7	LOS	Null	0	Yes, with normal breath
8	NLOS	Null	0	No
9	NLOS	Slow	1	No
10	NLOS	Fast	1	No
11	NLOS	Slow	2	No
12	NLOS	Fast	2	No
13	NLOS	Null	0	Yes, with turning over
14	NLOS	Null	0	Yes, with normal breath

## Data Availability

The data presented in this study are available on request from the corresponding author. The data are not publicly available due to privacy.
